# Are Pollution and Climate Change Potential Factors in the Development of Obsessive-Compulsive Disorder?

**DOI:** 10.31083/AP39005

**Published:** 2025-08-11

**Authors:** Luigi Attademo

**Affiliations:** ^1^Department of Mental Health, North West Tuscany Local Health Authority, 57023 Cecina, Italy

Environmental pollution and climate change have negative effects on mental health [1].

Little is known about whether exposure to environmental pollutants or climate effects contribute to or exacerbate psychopathologies like obsessive-compulsive disorder (OCD). The effects of pollution and climate stressors on OCD require further research. We do not know whether they are specific to this condition or are shared with other mental disorders [2, 3], but preliminary hypotheses can be made regarding factors that may explain these potential associations.

First, studies have found that extreme weather conditions and long-term exposure to different air pollutants, such as particulate matter (i.e., particulate matter 2.5 and 10 micrometers), nitrogen oxide (NO) and dioxide (NO_2_), carbon monoxide (CO), and sulphur dioxide (SO_2_), can exacerbate mental disorders [4, 5]. These pollutants may lead to systematic inflammation, oxidative stress, disruption of the blood-brain barrier, and alterations in brain function (for example, a hypothalamic-pituitary-adrenal axis disruption) [1], which could worsen obsessive-compulsive symptoms (OCS) or even possibly contribute to the development of OCD in vulnerable subjects. For example, increased levels of free oxygen radicals from environmental factors like pollution can have a negative impact on the human brain, inducing oxidative stress or damage. A significant relationship between oxidative stress and OCD has been found, suggesting an involvement of free radicals and of the antioxidant defence [6]. Evidence also exists that acute exposure to pollution is correlated with increased levels of OCS [7].

Second, exposure to environmental chemicals, such as pesticides, heavy metals (in particular, lead and mercury), food pollutants and microplastics, and endocrine-disrupting chemicals, may interfere with neurotransmitter systems and may affect brain structures involved in compulsive behaviors and anxiety regulation, potentially leading to or exacerbating OCS. Specifically, pollution may affect brain function by influencing serotoninergic and dopaminergic neurotransmitters [8], whose dysregulation is a core feature of OCD. Furthermore, the underlying cause of OCD has been suggested to possibly be the alteration of gut microbiota and consequent altered brain structures, due to the toxicological impact of environmental pollution [9].

Third, living in highly polluted environments or living in areas where climate change effects are more tangible, or in areas recently affected by a severe weather-related event, may also be negative contributing factors. In fact, these conditions may induce chronic stress, which could worsen pre-existing mental illnesses (including OCD). In addition, constant exposure to poor air quality or to climate threats may lead to higher anxiety levels; anxiety is a key component of OCD. In particular, the current issue of climate change and the perceived dangers associated with this phenomenon appear to directly and significantly affect the nature of the concerns experienced by subjects with the OCD checking subtype [10]. OCD is also likely to become more problematic when sufferers are exposed to pollution, because they always pay a lot of attention to their bodies and constantly think that they may become ill; thus, these feelings can lead to exacerbation of a pre-existing OCD condition [11].

Fourth, the biological mechanisms by which pollution and climate change influence the onset and exacerbation of mental disorders might also include gene expression [1]; subjects with a genetic predisposition to mental disorders may be more vulnerable to the effects of pollution or climate change on their mental well-being. Ambient environmental exposures during childhood and adolescence have been identified as a potential factor in the development of neurodevelopmental disorders (such as attention-deficit hyperactivity disorder, autism spectrum disorder, and OCD) through neuroinflammation, oxidative stress, and epigenetic dysregulation [12, 13].

And last, there is evidence that improving outdoor and indoor air quality may be associated with better outcomes in OCD. Green environments are associated with improved child brain development and mental health. Higher levels of greenspace around school and home may be associated with less OCS in primary school children [14]. The link between the lack of greenspace and the level or severity of OCS has been hypothesized to be through the stress produced by environmental factors such as air and noise pollution, and heat waves, which may all negatively affect the immune system.

In conclusion, both direct and indirect connections between pollution, climate change, and OCD can be hypothesized, although these connections are currently underexplored and require further investigation. However, no environmental risk factors have convincingly been associated with this mental disorder [15]. Indeed, most of the factors proposed seem not to be specific to OCD, but potentially related to other mental disorders, including anxiety disorders. Environmental and climate factors need to be recognized in the broader discussion of determinants of mental health now. In fact, mitigating environmental pollution and reducing the threats associated to climate change, have become crucial to reduce the risk of psychological disorders, including the risk of developing or exacerbating OCS.
